# Medicine-related problems: A recurrent issue among residents living in nursing homes

**DOI:** 10.3389/fphar.2022.978871

**Published:** 2022-08-29

**Authors:** Gereltuya Dorj, Renly Lim, Lisa Kalisch Ellett, Thu-Lan Kelly, Andre Andrade, Imaina Widagdo, Nicole Pratt, Rebecca Bilton, Elizabeth Roughead

**Affiliations:** Quality Use of Medicines and Pharmacy Research Centre, Clinical and Health Sciences, University of South Australia, Adelaide, SA, Australia

**Keywords:** adverse effects, prescription drug misuse, medication reconciliation, pharmacy services, medication therapy management, inappropriate prescribing, long-term care, medicine-related problems

## Abstract

**Aim:** To examine the incidence and nature of medicine-related problems over time experienced by nursing home residents.

**Method:** We analyzed records collected in the Reducing Medicine-Induced Deterioration and Adverse Events (ReMInDAR) trial. The trial pharmacists provided services to reduce medicine-induced deterioration and adverse reactions for residents every 8-weeks over a year. The problems identified by the pharmacists were documented in reports and subsequently classified independently by research pharmacists using the D.O.C.U.M.E.N.T system. The number and type of problems at each service and time to develop a new problem post first session were assessed. All analyses were performed using R software (Version 4.1.1).

**Results:** The cohort was 115 nursing home residents who received 575 services. In the 12-months, a total of 673 medicine-related problems or symptom reports were identified in 112 residents. Most residents (75%) experienced a new medicine-related problem by the fourth month post the first assessment. After the first session, the proportion of residents with a new medicine-related problem or symptom report declined at each repeated pharmacy session (59% at visit 2 vs. 28% at visit 6, *p* < 0.01).

**Conclusion:** Residents living in nursing homes frequently experience medicine-related problems. Our results suggest clinical pharmacist services performed every 4-months may have the potential to reduce the medicine-related problems in nursing homes.

## Introduction

Globally, there were 703 million people aged 65 years and older in 2019 (United Nations, Department of Economic and Social Affairs, Population Division, 2019). By 2050, one in six people will be aged over 65 years (United Nations, Department of Economic and Social Affairs, Population Division, 2019). Australia is no exception, with already 16% of the Australian population aged 65 years or older in 2018; the number is projected to increase to 23% by 2066 (Australian Institute of Health and Welfare, 2020). As of 2019, approximately 7% of Australians aged 65 years and older are living in residential aged care facilities (also known as a nursing home or long-term care facility) (Australian Institute of Health and Welfare, 2020).

People living in nursing homes are generally older, frailer, and have multiple co-morbidities that require the use of multiple medicines on a regular basis (Australian Institute of Health, 2012; Jokanovic et al., 2015). These characteristics combined with aging-related pharmacokinetic and pharmacodynamic changes (Mangoni and Jackson, 2004) result in a population at an elevated risk of medicine-related problems, medicine-induced deterioration, adverse health events, and death (Shah and Hajjar, 2012; Tamura et al., 2012; Onder et al., 2013). Medicine-induced deterioration is a cumulative effect of medicines encompassing symptoms such as cognitive and functional impairment, sedation or falls, loss of appetite, changes in urinary function and bowel function, changes in respiration, and changes in the activity of sleep patterns (Lim et al., 2019). Provision of medicine review by pharmacists is one method to reduce the risk of medicine-related harm and medicine-induced deterioration (Lee et al., 2019).

Pharmacist medicine reviews aim to improve and optimize medicine use, reduce harm, and improve patient outcomes (Australian Commission on Safety and Quality in Health Care, 2021). Pharmacist medicine reviews involve the assessment of medicine history, patient information, and clinical findings. The review considers individualized decisions on whether to continue, cease, or modify medicines and the review considers the interplay of therapeutic efficacy, comorbidities, compliance, medicine interactions, actual or potential adverse effects as well as assessing patients’ preferences and understanding of their illness (Zermansky et al., 2002).

In Australia, pharmacists are remunerated to formally perform collaborative medicine reviews in eligible older people living in nursing home, known as Residential Medication Management Review (RMMR) (Australian Commission on Safety and Quality in Health Care, 2021). Older people in nursing home can receive an RMMR if they meet any of the eligibility criteria, including newly admitted residents or existing residents who are currently experiencing medicine harm, or the referring medical practitioner confirms that there is a clinical need for an RMMR service (Australian Government-Department for Health, 2021). The funding rules allow a one-off medicine review visit once every 2 years or earlier if required, with a 2020 funding rule change allowing up to two follow-up visits within 9 months of the first visit (Pharmacy Programs Administrator, 2020).

Australian studies assessing the number of medicine-related problems identified at the time of medicine review in nursing home residents have found on average that there were three medicine-related problems per person (Pharmaceutical Society of Australia, 2019). Studies assessing the frequency of medicine-related problems in nursing home residents are often based on results from RMMRs and therefore represent prevalence estimates based on a single service conducted at a single point in time (Stafford et al., 2009; Nishtala et al., 2011; Kaur et al., 2012; Milos et al., 2013; Gheewala et al., 2014). Studies of pharmacist medicine reviews with follow-up or ongoing clinical medicine reviews are scarce; we located only four studies that had included multiple services or follow-up. None of these studies provided the frequency of medicine-related problems that occurred at each visit (Furniss et al., 2000; Patterson et al., 2010; Lapane et al., 2011; Frankenthal et al., 2014). As such, the ideal interval between pharmacist medicine reviews for older adults is unknown. We identified no studies that investigated how medicine-related problems emerge over time in nursing home residents.

To address this gap, this study aimed to assess the incidence and recurrence of medicine-related problems over time using data collected from participants who were enrolled in the Reducing Medicine-Induced Deterioration and Adverse Reactions (ReMInDAR) trial (Roughead et al., 2022).

## Materials and methods

The ReMInDAR trial was a randomized-controlled trial to reduce medicine-induced deterioration and adverse reactions in older adults living in nursing homes of Australia. Requirements to be enrolled in the study at baseline were if older adults were (United Nations, Department of Economic and Social Affairs, Population Division, 2019) aged 65 years and older (Australian Institute of Health and Welfare, 2020) using four or more medicines at the time of recruitment or taking more than one medicine with anticholinergic or sedative properties, and 3) had a frailty score ≤0.4 and were not-cognitively impaired (Roughead et al., 2022). Evidence suggests that frail older adults are at a higher risk of having adverse health outcomes (Shamliyan et al., 2013) when compared to non-frail individuals. To calculate frailty score, we used the frailty index, which is a validated instrument with 39-items encompassing multi-dimensional measures, allowing the assessment of physical, medical, psychological, and social contributors in older adults (Mitnitski et al., 2001; Mitnitski et al., 2005). The frailty score is a continuous score ranging from 0 to 1; a greater score indicates increased frailty. Furthermore, a frailty index score of 0.4 or greater is predictive of significant frailty, whereas a score of <0.25 is classified as non-frail (Rockwood et al., 2007; Theou et al., 2012; Widagdo et al., 2015).

Participants were randomly assigned to intervention and control groups. The intervention group received sessional pharmacist services for every 8 weeks over the 12-months intervention period. The intervention was focused on the early identification of potential harms from medicines and pharmacists used to validate tools to measure grip strength and cognition, as well as resident interview, patient history, and clinical care record review to identify potential harms from medicines (Roughead et al., 2022). More information regarding the ReMInDAR trial can be found elsewhere (Roughead et al., 2022). In this study, we analyzed data from participants who were enrolled in the intervention group.

### Collection of data

Pharmacist assessments, notes, actions, and recommendations were recorded by pharmacists at each session visit. In these visits, the trial pharmacist reviewed the same patient every 8 weeks, assessed their physical and cognitive performances, recorded new symptoms as identified by the resident or as documented in their care record, assessed changes to the medicine regimen, and identified actual or potential adverse medicine events (Roughead et al., 2022). The adverse events were assessed with a modified Naranjo method by a clinical panel. More information were published previously (Roughead et al., 2022).

The global pandemic restrictions due to Sars-Cov-2 have affected the final months (April–June 2020) of ReMInDAR trial, where some pharmacists’ sessions in some nursing homes had to be modified or stopped. The modifications allowed remote data review and interview by telehealth where possible. As a result, pharmacists were able to review medication charts and a summary of progress notes and adverse events remotely (Roughead et al., 2022).

### Assessment of medicine-related problems

#### Data classification

Medicine-related problems and symptoms that may be indicative of clinical deterioration or adverse effects were identified by the trial pharmacists and documented in the service report. The identified problems and symptoms were independently classified by research pharmacists using the categories proposed by the D.O.C.U.M.E.N.T classification (Williams et al., 2012). The D.O.C.U.M.E.N.T classification is a system to categorize medicine-related problems and clinical interventions performed in community pharmacy. As opposed to other methods available to assess medicine-related problems, the D.O.C.U.M.E.N.T classification has several advantages, including coding for activities intended to resolve medicine-related problems, assessing the impact of intervention and clinical significance, and it as reported to be well-suited to use in the Australian community pharmacy environment (Williams et al., 2012).

Categories of the D.O.C.U.M.E.N.T classification include inappropriate medicine selection, over-dose or under-dose prescribed, compliance, undertreated, need for monitoring, need for education or information, toxicity, or adverse reaction (Williams et al., 2012). Clinical problems that do not fit under any other category are coded as not classifiable (Williams et al., 2012).

Because of the nature of our study, which focused on identifying signs and symptoms of adverse effects, we created an additional category: symptom reports. Symptoms classified in this category frequently included pain, cognitive decline, sedation, and weight gain. Pharmacists indicated when they observed these problems and whether they thought causality to a medicine was possible. To indicate the causality, pharmacists reported that additional information was required and further assess residents. Symptoms were extracted as verbatim text by one research pharmacist (GD) and visualized using the R package “wordcloud” (Oesper et al., 2011).

One research pharmacist (GD) classified all records documented by the ReMInDAR pharmacists. Validation of the classification was performed by a second independent research pharmacist (RL) on a randomly selected sample of 108 (19%) pharmacist services (*n* = 575). Cohen’s kappa to quantify the level of agreement between identification and classifications was used (Cohen, 1960). The computation of kappa values was performed using the vcd package for open-source R Studio Version 1.2.1335 (R Development Core team, 2009) (Friendly and Meyer, 2015). The level of agreement was high with kappa = 0.85 (95% CI, 0.76–0.95, *p* < 0.0001) (Cohen, 1960).

We estimated the proportion of participants who had a problem at each session (Eq. 1)

proportion of people with medicine-related problem=Number of people with medicine-related problem/Total number of peopel enrolled and visited by a pharmacist (1)

### Identification of new medicine-related problems

Because pharmacists were visiting residents every 8 weeks, we were able to investigate when residents developed new medicine-related problems subsequent to the first session. In the analysis of new medicine-related problems, we investigated the proportion of people identified with new problems at each session. A new medicine-related problem was defined as any medicine-related problem that was not identified at the first visit; the first pharmacist visit was the reference point. Problems identified in the subsequent sessions were analyzed if they were not documented in the previous session.

For example, a patient has received a clinical pharmacist service six times throughout the trial. No problems were identified at the first session. However, between the second session and third session, the patient had a stroke and was no longer able to swallow their medicines. This was assessed as a “new medicine-related problem” that was recorded at the third session.

The proportion of people identified with new medicine-related problems was based on the following equation:

proportion of people with new medicine-related problem=Number of people with new medicine-related problem/Total number of peopel enrolled and visited by a pharmacist (2)

### Analysis of time to new medicine-related problem

To estimate the time to the first new medicine-related problem after the first session, we included timelines for individual sessions by pharmacists.

### Statistical analysis

Continuous variables, including the proportion of patients identified with problems at each session, were compared using the t-test. To estimate the probability to experience a new medicine-related problem, a time-to -event analysis using the Kaplan–Meier method was undertaken (Kaplan and Meier, 1958), censoring for death and end of study.

Descriptive results are provided as median and standard deviation (SD), unless otherwise stated. A *p* value less than 0.05 was considered statistically significant. All estimates were computed using the R software (Version 4.1.1) and the R packages “survminer,” “stats,” and “wordcloud” were used (Kaplan and Meier, 1958; Oesper et al., 2011; Team, 2013; Kassambara et al., 2017).

## Results

### Baseline characteristics of participants

The cohort for this study was 115 participants who received 575 pharmacist services delivered by 29 pharmacists. At baseline, the mean age of participants was 85 years (SD = 7.4) and most were women (*n* = 76, 66.1%).

The mean number of unique medicines per participant at baseline was 15.1, and the participants used, on average, two medicines with anticholinergic or sedative properties (Table 1).

**TABLE 1:** Baseline characteristics of participants enrolled in the intervention group.

Characteristics	Measurement (n, SD)
Age, years	85 (7.4)
Gender: Female	76 (66.1%)
Mean number of unique medicines	15.1 (5.7)
Mean number of anticholinergic or sedative medicines	2 (1.5)

### The incidence and nature of medicine-related problems

Over 12 months, residents had on average six medicine-related problems or symptom reports identified by pharmacists. At each session, over 30% of residents had a symptom report identified and 40% of residents had a medicine-related problem other than a symptom report (Table 2). Excluding symptom reports, a total of 277 medicine-related problems were recorded, with a median of three per person (SD = 1.3). In the 12-months trial, three people had no problems. When looking at the distribution of problems by different sessions, the proportion of residents experiencing no medicine-related problems increased over time (21% had no problems at session 1 rising to almost 40% with no problems at session 6). Consistent with the service design, the decline in participants with toxicity or adverse events was the highest, from 22% at session 1 and dropping down to 11% at session 6 (Table 2).

**TABLE 2:** Number of residents identified with medicine-related problems, by session and by problem type.

Problem Type	Session 1	Session 2	Session 3	Session 4	Session 5	Session 6
	*n* = 115	*n* = 109	*n* = 106	*n* = 9	*n* = 91	*n* = 57
	*n*, %	*n*, %	*n*, %	*n*, %	*n*, %	*n*, %
Toxicity or adverse reaction	25, 22%	18, 17%	19, 18%	11, 11%	9, 10%	6, 11%
Education or information	22, 19%	18, 17%	17, 16%	22, 23%	12, 13%	8, 15%
Over- or underdose	15, 13%	10, 9%	17, 16%	17, 18%	13, 14%	5, 9%
Drug selection	14, 12%	9, 8%	9, 8%	5, 5%	2, 2%	2, 4%
Compliance	8, 7%	5, 5%	1, 1%	5, 5%	2, 2%	3, 5%
Monitoring	6, 5%	6, 6%	11, 10%	8, 8%	6, 7%	3, 5%
Symptom report	40, 35%	43, 39%	31, 29%	33, 34%	21, 34%	17, 30%
No problems	24, 21%	25, 23%	27, 25%	27, 28%	32, 35%	22, 39%

### Nature of symptom reports

**FIGURE 1:**
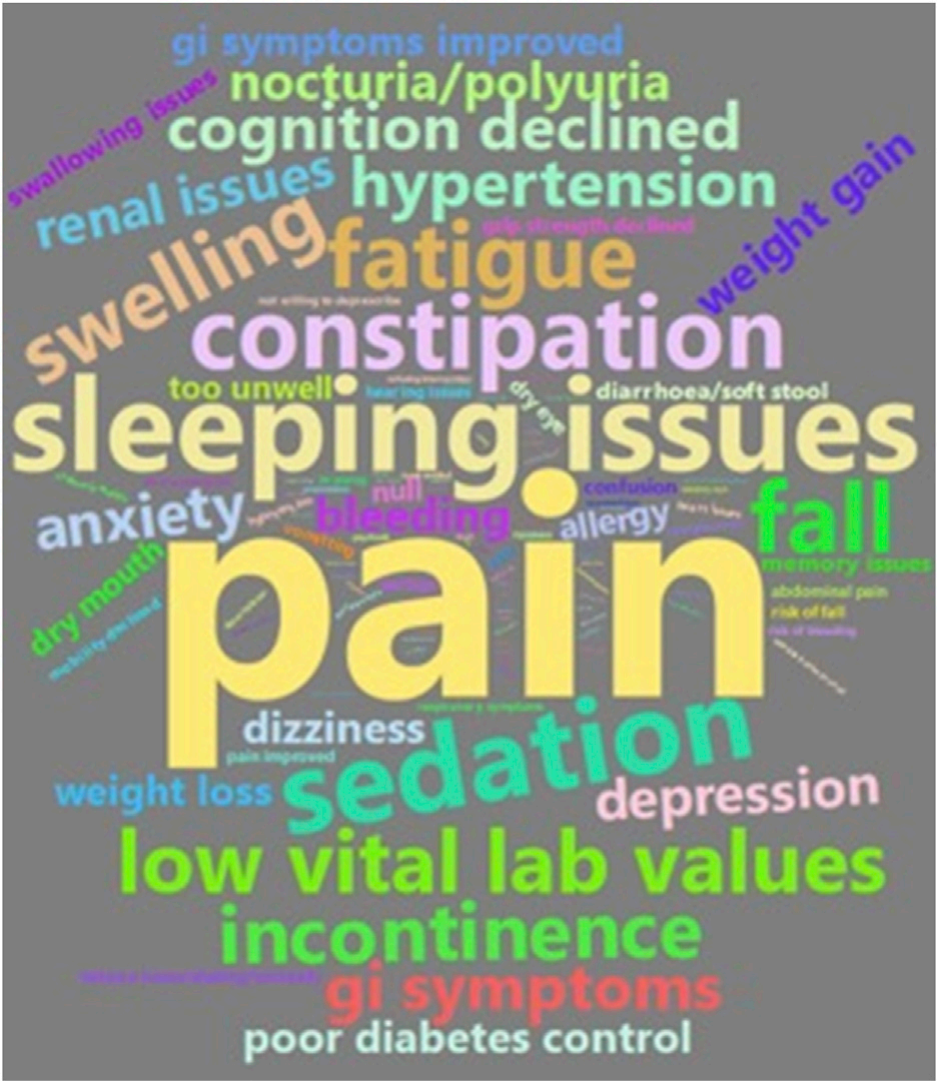
Nature of symptom related reports documented by the pharmacists..

There were 396 symptom-related reports among 103 participants (90%) Most reports were pain (*n* = 79), followed by sleeping issues (*n* = 39), sedation (*n* = 29), and constipation (*n* = 25) (Figure 1).

### New and ongoing medicine-related problems or symptom reports

**FIGURE 2:**
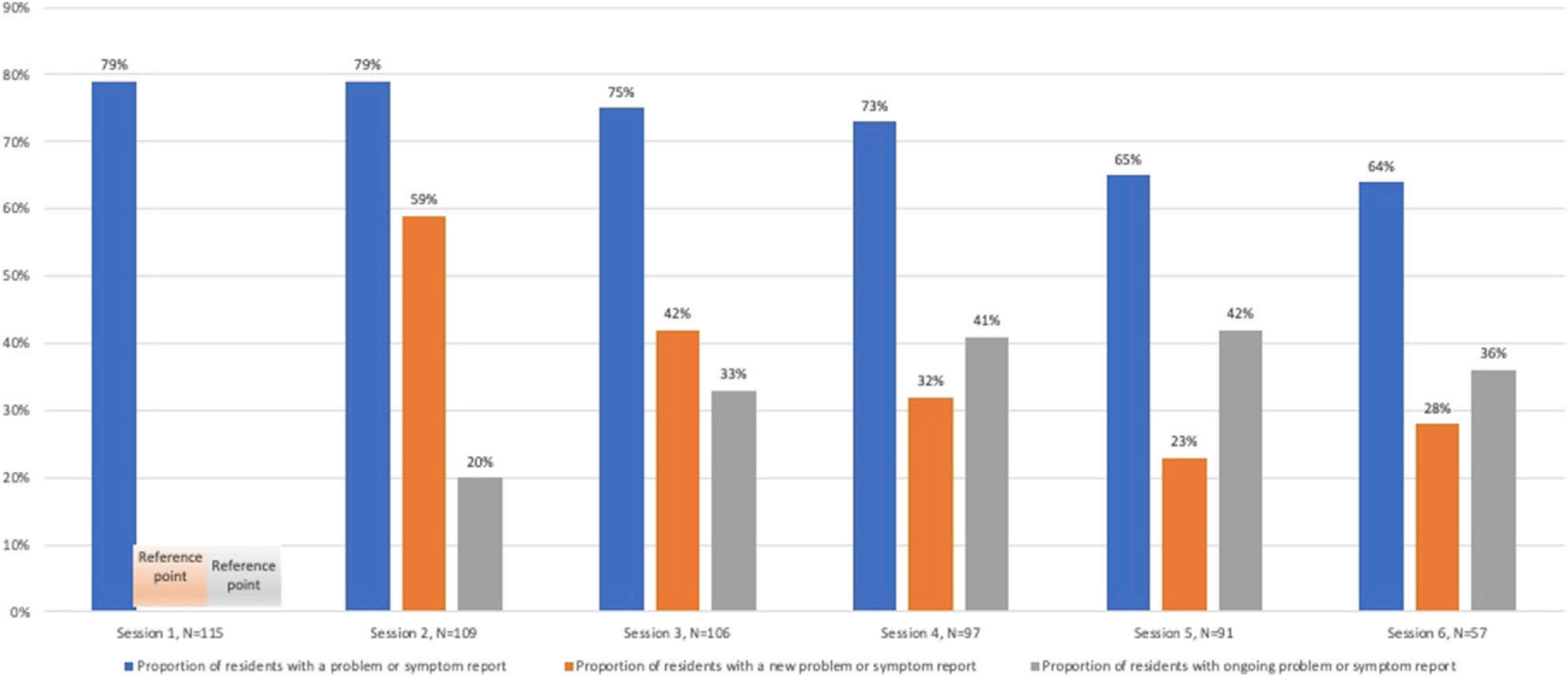
Proportion of residents with new and ongoing medicine-related problems or symptoms at each session.

In the trial, 71% of residents had a new medicine-related problem identified within the period after the first pharmacist session (∼10 months). The median number of new medicine-related problems over the 10 months was two (SD = 1.4). There was a trend towards less new medicine- related problems or symptoms over time (*p* < 0.01). Pharmacists documented ongoing symptoms or problems in up to 40% (Figure 2).

Review of the pharmacist notes demonstrated that it may take several visits to resolve a medicine-related problem in residents. In the example below, we provide a case where the ReMInDAR pharmacist required four sessions to identify and resolve a medicine-related problem.

At the first visit, the ReMInDAR pharmacist noted that the resident had bright red blood in their stool. The pharmacist notified the nurse and doctor and a review of apixaban (5 mg twice daily). During subsequent visits (Australian Institute of Health, 2012; Australian Institute of Health and Welfare, 2020), the resident was still experiencing blood in their stool. The pharmacist continued monitoring the resident and considering the resident’s age (>80 years old), weight (<60 kg), laboratory results (CrCl = 0.66 mg/dl), contacted the doctor again recommending to reduce the dose of apixaban. By session 4, the doctor finally accepted to trial reducing apixaban from 5 mg twice daily to 2.5 mg twice daily. The resident informed the pharmacist at session 5; the blood in the stool had stopped.

### Probability to experience a new medicine-related problem or new symptom, post first session

**FIGURE 3:**
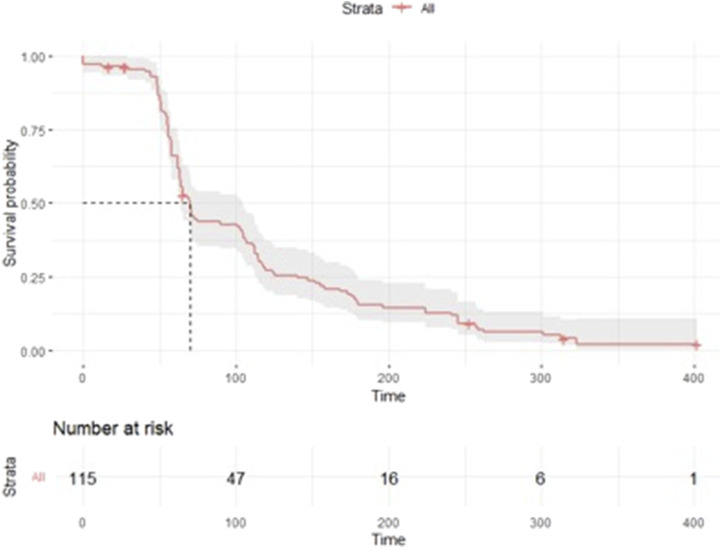
Time to experience a new medicine-related problem or symptom.

We analyzed the cumulative probability to experience a new medicine-related problem or symptom after session 1 and found that 59% of participants had a new medicine-related problem or a new symptom report by the next session (8 weeks) and 75% had a new problem by the subsequent session (∼16 weeks), and 90% by the third session (∼24 weeks) (Figure 3).

## Discussion

### Medicine-related problems

We investigated the frequency and nature of the medicine-related problems in nursing home residents over a 12-month period. In our study, most residents had at least one medicine-related problem, the number of medicine-related problems, or symptom reports was six per person; when limited to medicine-related problems only, it was three per person. This study is the first study to look at the development of new medicine-related problems over time, finding that new problems emerge within 8–16 weeks for the majority of the studied population. The proportion of residents with new medicine-related problem declined over the trial period (39% vs. 25%, *p* = 0.05). The reduction in medicine-related problems over time is likely due to the repeated pharmacist visits in the ReMInDAR intervention, where pharmacists consistently monitored residents for adverse effects of medicines and intervened to resolve them. Previous Australian studies in nursing homes have looked at medication-related problems from a single medication review (Pharmaceutical Society of Australia, 2019), where reviews are usually at least 12-months apart, reporting the average number of medicine-related problems being three per person (Pharmaceutical Society of Australia, 2019).

### Symptom reports

Symptom reports were recorded in over 30% of residents at each session, with common symptoms reported as pain, cognitive decline, sedation, and weight gain. Our intervention focused on reducing medicine harms with pharmacists encouraged to assess patients for adverse effects with the help of validated tools including grip strength and Montreal Cognitive Assessment (Roughead et al., 2022). This intervention may have supported the high number of symptom reports documented by the pharmacists. Identification of symptoms, such as drowsiness or change in cognition, is important for enabling the prevention of the consequential, more serious medicine-induced harms of falls and delirium (Inouye et al., 2007). Our review did not locate any repeated pharmacist service trials that reported how pharmacists documented and assessed symptom reports in nursing home residents. Further studies could investigate the utilization of clinical notes, including the symptom reports in nursing home residents to better identify people experiencing medication-related deterioration or harms.

### Frequency of medicine reviews

Only four prior studies tested clinical medicine reviews involving multiple services in nursing home residents, but none reported how problems emerge over time (Furniss et al., 2000; Patterson et al., 2010; Lapane et al., 2011; Frankenthal et al., 2014). A previous study in the United kingdom provided a single medicine review by a pharmacist with one follow-up visit but problems by visit were not reported (Furniss et al., 2000). Frankenthal et al. provided a pharmacist service for nursing home residents with two visits over a 6-month period (Frankenthal et al., 2014). While the number of medicines was reduced at the end of the study (*p* < 0.001), how problems emerged at each visit was not reported (Frankenthal et al., 2014). A randomized clinical trial in 2010 assessed the effectiveness of a monthly pharmacist service delivered for nursing home residents (Patterson et al., 2010). In the trial, pharmacists focused on psychoactive medicines and provided reviews based on a previously published algorithm. After 1 year, the proportion of residents taking inappropriate psychoactive medications in the intervention group (25/128, 19.5%) was much lower than in the control group (62/124, 50.0%) (OR = 0.26, 95% CI 0.14–0.49); however, the need for the frequency of the service was not assessed. Finally, a US-based study trialed a pharmacist intervention using an algorithm generated from clinical care records of nursing home residents (Geriatric Risk Assessment MedGuide) (Lapane et al., 2011). Pharmacists visited nursing home every month, and medicine reviews were mandated 1–3 times a year. The residents in control group received a similar number of interventions as the intervention group. Therefore, the study assessed the additional benefits of the algorithm and not really the pharmacist service (Lapane et al., 2011). None of these studies reported the change in medicine-related problems that occurred at each visits.

Medicine reviews are recognized as a key intervention to reduce the risk of medicine-related problems in nursing home residents. Currently, an Australian resident entering a nursing home is allowed to receive a formal RMMR service only once every 2 years, with up to two follow-ups within 9 months of the first service (Pharmacy Programs Administrator, 2020). Our findings suggest that more regular pharmacist-led services, performed at least every 4 months, have the potential to reduce the number of medicine-related problems in nursing home residents over time (Thiruchelvam et al., 2017).

## Limitation

The ReMInDAR trial was affected by COVID-19. Pharmacists were scheduled to visit their participants every 8 weeks over a 12-month period. However, due to restrictions during COVID-19 and unavailability of participants, some pharmacist visits had to be undertaken remotely or were delayed (*n* = 53, 9%), while some were unable to be performed (*n* = 35, 6%). Hence, some symptoms and medication-related problems might have been under-reported at visit 6. However, the frequency of problems and symptoms was similar between visit 5 (where COVID-19 had little impact) and visit 6. In addition, the majority of pharmacists’ visits (85%) were performed as planned; thus, our findings may represent the current extent of the problem among older people living in nursing homes.

A relatively large number of pharmacists (*n* = 29) engaged in the ReMInDAR trial who assessed residents may have created some bias regarding their judgment. However, the ReMInDAR trial pharmacists reviewed the same patient at every visit consulting and reporting any progress with treating doctor and nursing home staff.

The results were reliant on the completeness of documentation during the pharmacist service. While we cannot ascertain completeness, we were trialing a new intervention, and ongoing pharmacist support and training, including on-site peer visiting, was provided throughout the trial to assist pharmacists with documentation.

Finally, the incidence and frequency of medicine-related problems were identified based on the population aged 65 years old and older living in nursing homes of Australia. However, our findings may be applicable to other countries as there is evidence that medicine-related problems are prevalent in this population living in similar settings.

## Conclusion

In summary, we described the nature and frequency of medicine-related problems that occurred over time in nursing home residents. We found that medicine-related problems arose throughout the year as residents’ health and medicines changed. Our results suggest that pharmacist review every 4 months may be warranted to ensure the quality use of medicines in nursing homes.

## Data Availability

The raw data supporting the conclusions of this article will be made available by the authors without undue reservation.
